# Long-Term Outcome After Adoptive Immunotherapy With Natural Killer Cells: Alloreactive NK Cell Dose Still Matters

**DOI:** 10.3389/fimmu.2021.804988

**Published:** 2022-01-31

**Authors:** Sarah Parisi, Loredana Ruggeri, Elisa Dan, Simonetta Rizzi, Barbara Sinigaglia, Darina Ocadlikova, Andrea Bontadini, Valeria Giudice, Elena Urbani, Sara Ciardelli, Chiara Sartor, Gianluca Cristiano, Jacopo Nanni, Letizia Zannoni, Gabriella Chirumbolo, Mario Arpinati, Russell E. Lewis, Francesca Bonifazi, Giovanni Marconi, Giovanni Martinelli, Cristina Papayannidis, Stefania Paolini, Andrea Velardi, Michele Cavo, Roberto M. Lemoli, Antonio Curti

**Affiliations:** ^1^ IRCCS Azienda Ospedaliero-Universitaria di Bologna, Istituto di Ematologia “Seràgnoli”, Bologna, Italy; ^2^ Division of Hematology and Clinical Immunology, Department of Medicine, University of Perugia, Ospedale Santa Maria della Misericordia, Perugia, Italy; ^3^ Department of Experimental, Diagnostic and Specialty Medicine, University of Bologna, Bologna, Italy; ^4^ Blood Transfusion Department, Santa Maria degli Angeli Hospital, Pordenone, Italy; ^5^ Immunohematology Service and Blood Bank, University Hospital S.Orsola-Malpighi, Bologna, Italy; ^6^ Department of Medical and Surgical Sciences, University of Bologna, Bologna, Italy; ^7^ Istituto Scientifico Romagnolo per lo Studio e la Cura dei Tumori (IRST) IRCCS, Meldola, Italy; ^8^ Clinic of Hematology, Department of Internal Medicine (DiMI), University of Genoa, Genoa, Italy; ^9^ IRCCS Policlinico S. Martino, Genoa, Italy

**Keywords:** acute myeloid leukemia, natural killer cells, adoptive immune therapies, alloreactivity, cell dose

## Abstract

Recently, many reports were published supporting the clinical use of adoptively transferred natural killer (NK) cells as a therapeutic tool against cancer, including acute myeloid leukemia (AML). Our group demonstrated promising clinical response using adoptive immunotherapy with donor-derived alloreactive KIR-ligand-mismatched NK cells in AML patients. Moreover, the antileukemic effect was correlated with the dose of infused alloreactive NK cells (“functional NK cell dose”). Herein, we update the results of our previous study on a cohort of adult AML patients (median age at enrollment 64) in first morphological complete remission (CR), not eligible for allogeneic stem cell transplantation. After an extended median follow-up of 55.5 months, 8/16 evaluable patients (50%) are still off-therapy and alive disease-free. Overall survival (OS) and disease-free survival (DFS) are related with the dose of infused alloreactive NK cells (≥2 × 10^5^/kg).

## Introduction

Natural killer (NK) cells are lymphocytes expressing CD56 or CD16 antigens on their surface and lacking CD3. NK cells play a central role in controlling tumor development, and their activity depends on a number of receptors with activating and inhibitory activity. In particular, receptors named “killer cell immunoglobulin-like receptors” (KIRs) act as inhibitors and are able to bind MHC class I alleles (1, 2). The first data demonstrating a beneficial effect of haploidentical KIR-ligand-mismatched NK cells in leukemia control were derived from haploidentical T-cell-depleted allogeneic stem cell transplantation (SCT) (3–5). Outside the transplant setting, Miller and colleagues (6) first demonstrated that acute myeloid leukemia (AML) patients could be safely and effectively treated with adoptive immunotherapy with haploidentical NK. The authors retrospectively demonstrated that the presence of KIR-L mismatch between donor and recipient was significantly related with a better response in preventing leukemia relapse.

Based on this seminal work, our group analyzed the results of adoptive immunotherapy with donor-derived highly purified KIR-ligand-mismatched, haploidentical NK cells in a cohort of relapsed or refractory, adult, high-risk, AML patients in a phase I study (7). NK cell adoptive immunotherapy was well tolerated, and no graft-versus-host disease (GVHD) occurred. Moreover, we showed that it was possible to track donor-versus-recipient alloreactive NK cells *in vivo* after infusion. Adoptively transferred NK cells resulted to be alloreactive against the leukemic cells of the recipient. In a subsequent study, we enrolled 17 elderly AML patients, in first morphological CR, unfit for allogeneic stem cell transplantation (ASCT) (8). Again, tolerance to the procedure was optimal. Regarding early clinical results, among 16 patients evaluable for response, 9 were alive and disease-free after a median follow-up of 22.5 months (range, 6–68 months). Interestingly, the frequency of donor alloreactive NK cells was shown to be related with the response and duration of the response. Moreover, we observed that relapse occurrence was significantly related to the percentage of donor-derived alloreactive NK cell clones before (*p* = 0.003). Indeed, it was possible to identify a functional dose of 2 × 10^5^/kg alloreactive NK cells that resulted to be predictive of response in our cohort of patients (9). On the basis of these findings, we decided to perform a long-term analysis of our original data after a median follow-up of 55.5 months (range, 6–125 months).

## Results and Discussion

Median age at enrolment was 64 years (range, 53–73 years). The characteristics of the patients were previously described (8). Haploidentical KIR-L-mismatched donors were selected if at least one allele mismatch at a class I locus among the following ones was present: HLA-C alleles with Asn77-Lys80, HLA-C alleles with Ser77-Asn80, and HLA-Bw4 alleles. Haploidentical NK cells were purified from a steady-state large volume leukapheresis product. We proceeded with NK cell purification only if the donor leukapheresis product contained 10 × 10^6^ NK cells/kg. Indeed, we evaluated the repertoire of KIR-L-mismatched donor alloreactive NK cell at least 30 days prior to steady-state leukapharesis to assess the functional cell dose, since the NK repertoire is stable with time (see below).

For NK cell purification, a maximum number of 40 × 10^9^ total PB mononuclear cells obtained from steady-state large volume leukapheresis were selected by the CliniMACS device (Miltenyi Biotec, Bergisch Gladbach, Germany). Cells were incubated with MACS colloidal superparamagnetic microbeads conjugated to monoclonal mouse anti-human CD3 and anti-human CD56 antibodies (Miltenyi Biotec) in two steps: first CD3^+^ T cells were depleted and then CD56^+^ NK cells were positively selected under good manufacturing product (GMP) conditions. Purified CD56^+^CD3^−^ NK cells were cryopreserved. In particular, in order to perform cryopreservation and storage in liquid nitrogen, NK cells were diluted in homologous plasma and DMSO, then cooled in a programmed biological freezing unit (CRYO 10-16, Planer, USA) and stored in the gas phase of a liquid nitrogen tank until thawing. Before cryopreservation, the following tests were performed on the NK cell product: sterility testing (fungal and bacterial), viability, assessment of nucleated cell dose, extensive phenotyping to determine the purity of NK cells in the final cell product, and the presence of residual B and T cells and monocytes. Subsequently, we determined the number of alloreactive NK cells, on the basis of the frequency of alloreactive NK cells. In case that the minimum collected cell dose of 2 × 10^5^ alloreactive NK cells/kg was not obtained after a single leukapheresis, it was possible for donors to undergo a second PB collection within 30 days from the first one. Since the NK repertoire is known to be stable with time, the evaluation of KIR-L-mismatched donor alloreactive NK cell repertoire was conducted at baseline, in order to determine the functional cell dose. Briefly, we were able to document the frequency of alloreactive clones through the generation of large numbers of donor alloreactive NK clones and cytotoxicity assays against recipient target cells performing direct functional assessment of donor NK cell repertoire. The results of this analysis were used after NK cell collection and purification to determine whether the minimally accepted functional cell dose of alloreactive NK cells (2 × 10^5^ alloreactive NK cells/kg) was obtained and to make correlation with clinical response (8).

All patients received a lympho-depleting chemotherapy regimen with fludarabine 25 mg/m^2^ (day −7 to −3) and cyclophosphamide 4 g/m^2^ (day −2), followed by NK cell infusion (day 0) and by IL-2 injection (10 × 10^6^ IU/day, 3 times weekly for 2 weeks: 6 doses total). Among 16 evaluable patients, 8 (50%) were alive and disease-free, after a prolonged follow-up (55.5 months; range, 6–125 months). Eight patients relapsed after a median time of 9 months from CR (range, 5–51 months). Among the relapsed patients, three remained in CR after NK cell adoptive immunotherapy at 15, 24, and 51 months, respectively. The patient who relapsed after 51 months was treated with a second NK cell infusion and obtained a second CR. Intriguingly, two patients who were infused with positive measurable residual disease (MRD) achieved MRD clearance after one NK cell infusion. Based on these data, 11 of 16 (69%) patients were responders, whereas 5 of 16 (31%) were non-responders.

**Figure 1:**
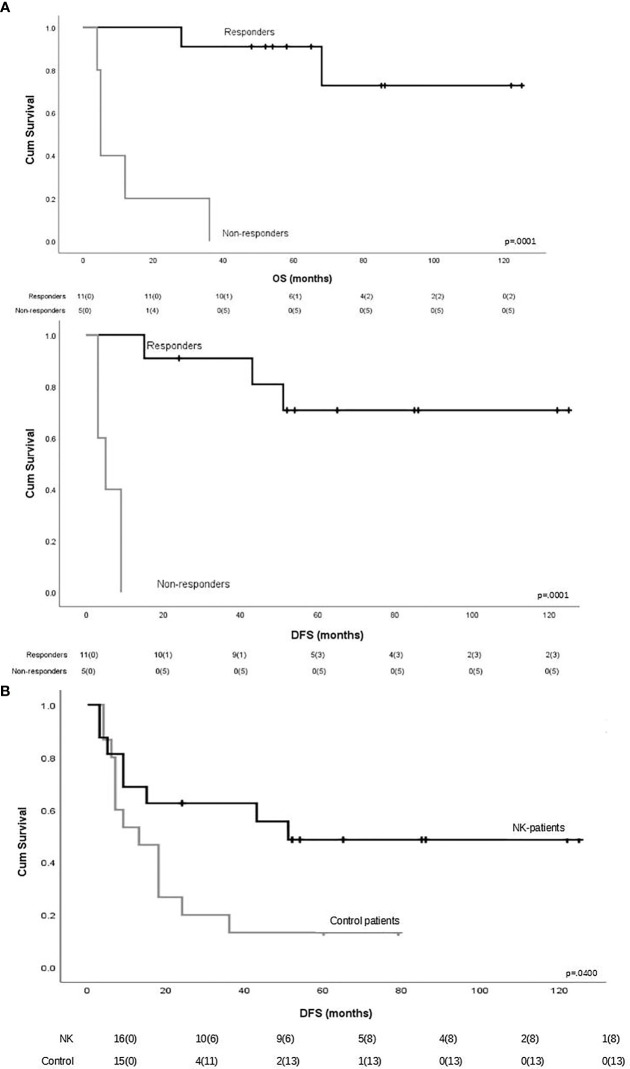
**(A)** Impact of response to natural killer (NK) cell immunotherapy on long-term outcome in terms of overall survival and disease-free survival. Responders were defined as those patients who maintained CR for at least 6 months after NK cell infusion (*n* = 11). Patients maintaining CR after NK cell infusion had a significantly better outcome as compared with non-responder patients. **(B)** Long-term disease-free survival in patients who received or did not receive NK immunotherapy.

**Figure 2:**
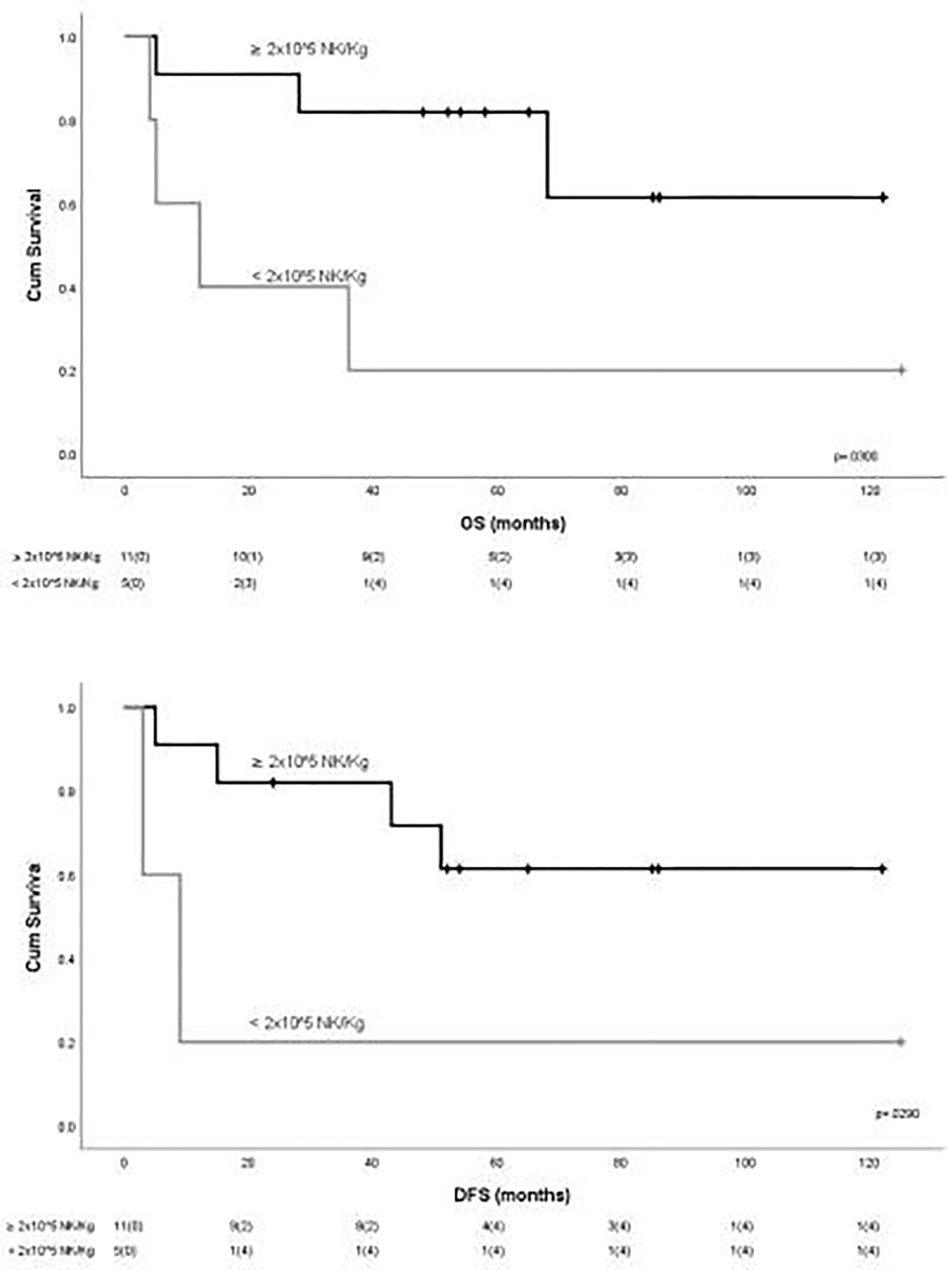
Outcome of patients according to the “functional dose” of infused NK cells. Statistically significant better outcome in terms of OS and DFS was observed for patients receiving more than 2 × 10^5^ NK/kg.

To evaluate the impact of response to NK cell immunotherapy on long-term DFS and OS, we compared the clinical outcome of responder versus non-responder patients. We considered as responders those patients who maintained in CR for at least 6 months after NK cell infusion, which is the median time to relapse in the control group. As shown in **Figure 1A**, patients who obtained a response to NK cell infusion had a significantly better outcome as compared with non-responding patients. We here compare these long-term clinical results with the outcome of patients from the same historical control cohort we analyzed in our previous report (8), treated with the same induction/consolidation chemotherapy, who did not receive NK immunotherapy. In the control group, 14 out of 15 patients (93%) relapsed with a median of 11 months (range 3–79). The difference in terms of DFS between the two groups of patients is statistically significant (**Figure 1B**). The outcome of patients was then analyzed according to the previously described “functional dose” of infused alloreactive NK cells (i.e., 2 × 10^5^/kg) (8). Indeed, long-term analysis confirmed our previous data, supporting the view that patients who had received more than 2 × 10^5^/kg alloreactive NK cells may have significantly improved OS and DFS (**Figure 2**). With the limitation of the low number of patients, these data show the long-term correlation between response to NK cell immunotherapy and the frequency of infused alloreactive NK cells.

**Figure 3:**
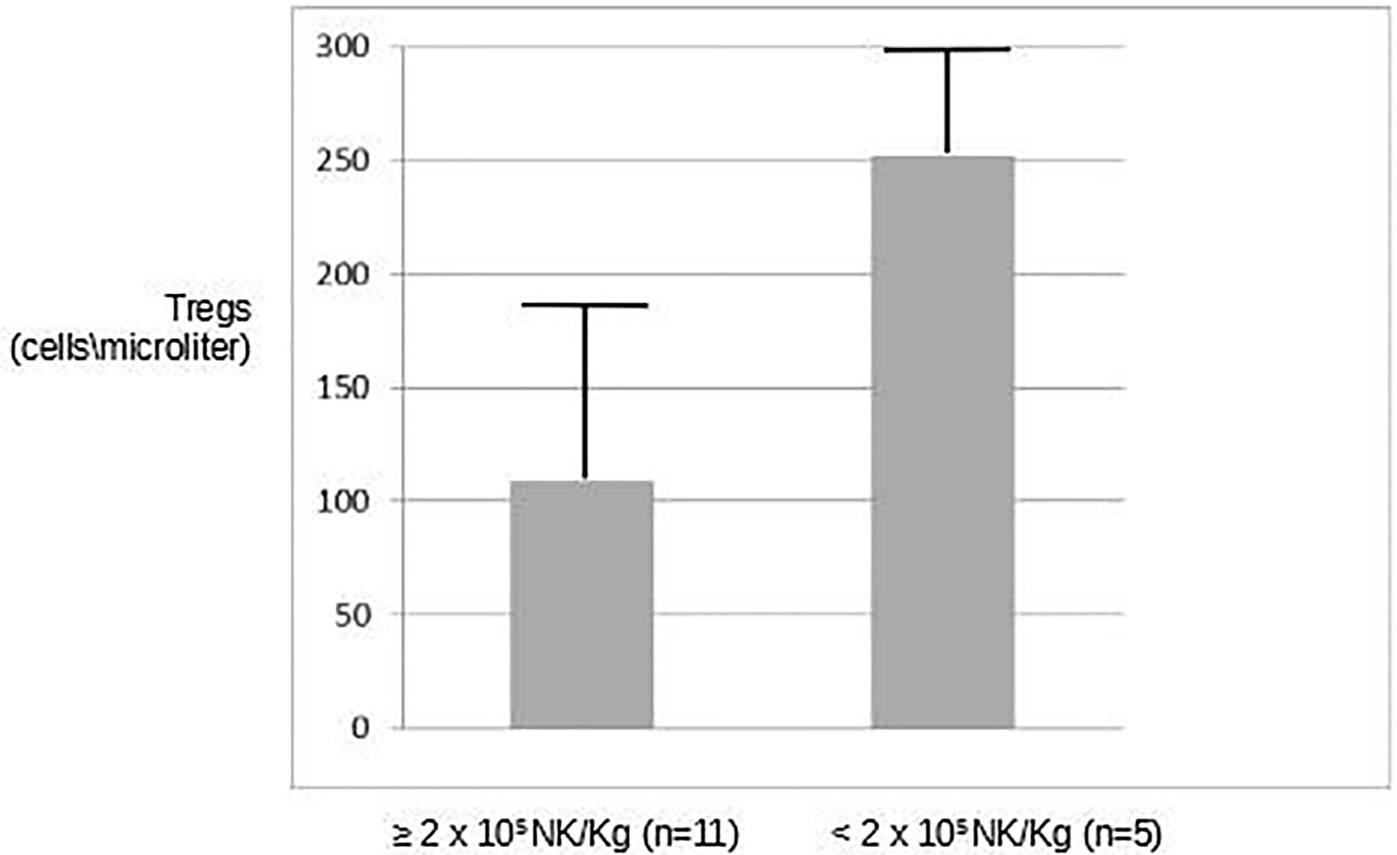
Correlation between long-term clinical outcome and frequency of Tregs after infusion. A lower number of Tregs were observed in patients who had received more than 2 × 10^5^/kg alloreactive NK cells.

Previous reports have suggested the detrimental effects of regulatory T cells on the effector function of NK cells infused in cancer patients as a means of adoptive immunotherapy (6) and negatively correlated Tregs number with clinical response. Interleukin-2, which is known to potently increase the frequency and the immunosuppressive effects of Tregs (10), is part of our *in-vivo* strategy to augment the activity of infused NK cells. Thus, we sought to analyze the correlation between long-term clinical outcome and the frequency of Tregs after infusion. Even though the small number of patients did not allow to obtain conclusive results, we detected a lower number of Tregs in patients who had received more than 2 × 10^5^/kg alloreactive NK cells (105 Tregs/µl ± 77 vs. 250/mmc ± 48, respectively (**Figure 3**).

In conclusion, with an extended follow-up of 55.5 months, we point out that an adequate dose of adoptively transferred alloreactive NK cells has the potential to induce a durable control of AML in elderly patients who are unfit for allogeneic stem cell transplantation. Furthermore, the present long-term analysis reinforces the concept that the frequency of NK cells in the infused product may be considered a marker with a predictive value for the clinical outcome (11). The possibility of infusing a number of functionally active NK cells is likely to have a greater impact on the efficacy of NK infusion than simply enumerating the number of total NK cells. Further studies addressing this specific point are currently ongoing at our centers.

## Data Availability Statement

The raw data supporting the conclusions of this article will be made available by the authors, without undue reservation.

## Ethics Statement

The studies involving human participants were reviewed and approved by Comitato Etico Area Vasta Emilia Centro. The patients/participants provided their written informed consent to participate in this study.

## Author Contributions

SaP and AC wrote the article and analyzed the data. LR, EU, ED, SR, BS, DO, SC, and GaC were involved in the cell processing and laboratory analysis. CS, GiC, JN, and LZ collected the data. AB, VG, MA, FB, MC, RML, CP, StP, and AV reviewed the work. REL performed the statistical analysis. All authors contributed to the article and approved the submitted version.

## Conflict of Interest

The authors declare that the research was conducted in the absence of any commercial or financial relationships that could be construed as a potential conflict of interest.

## Publisher’s Note

All claims expressed in this article are solely those of the authors and do not necessarily represent those of their affiliated organizations, or those of the publisher, the editors and the reviewers. Any product that may be evaluated in this article, or claim that may be made by its manufacturer, is not guaranteed or endorsed by the publisher.
